# Uncovering hidden threats: prevalence, antibiotic resistance and virulence gene profiles of *Escherichia coli* strains isolated from Testudines and their aquatic habitats

**DOI:** 10.1007/s11033-025-10857-w

**Published:** 2025-08-02

**Authors:** Lungile N. Mlangeni, Tsepo Ramatla, Cormac Price, Oriel Thekisoe, Che Weldon

**Affiliations:** 1 Unit for Environmental Sciences and Management, North-West University, Potchefstroom, 2531 South Africa; 2 Centre for Applied Food Safety and Biotechnology, Department of Life Sciences, Central University of Technology, 1 Park Road, Bloemfontein, 9300 South Africa

**Keywords:** *E. coli*, Testudines, Antimicrobial resistance, Virulence genes, Environmental bacteria

## Abstract

**Background:**

The gut microbiota of Testudines is fundamental to their digestion and overall health, yet remains a poorly investigated area in their biology, particularly in wild freshwater turtle (terrapins) and tortoise populations within South Africa. This study investigated the occurrence, diversity, virulence genes and antibiotic resistance of *Escherichia coli* isolated from Testudine gut microbiota and sediments at Timbavati Private Nature Reserve, South Africa.

**Methods and results:**

Cloacal swab samples were collected from 36 wild Testudines and 20 sediment samples from temporary and permanent water bodies. Presumed *E. coli* isolates were confirmed by polymerase chain reaction (PCR) targeting the *β*-D glucuronidase (*uidA*) gene and further validated through *16 S rRNA* gene sequencing. Phenotypic antibiotic resistance was evaluated with the Kirby-Bauer method, whilst resistance and virulence genes were identified using PCR assays. *E. coli* was detected in 54 (62%) of 87 isolates (23 Testudines and 31 sediments), confirmed by *uidA* PCR assay. Detected virulence genes included *eaeA* (42%), *virF* (22%), *stx1* (16%), and *stx2* (3%), and isolates exhibited resistance to erythromycin (53%), cephalothin (48%), and spectinomycin (40%). Resistance genes such as *mcr-4* (70%), *bla*_SHV_ (46%), *bla*_TEM_ (64%), *mcr-1* (42%), *qnrA* (16%), *mcr-2* (22%), *qnrD* (11%), and *tetW* (2%) were also detected.

**Conclusions:**

This study demonstrates that wild Testudines harbour *E. coli* in their gut and that it also occurs in their surrounding environment, with notable antibiotic resistance and virulence potential. The findings underscore the complexity of host-microbial interactions and the influence of environmental and host factors on microbial diversity, informing potential conservation and health management strategies for these reptilian species.

## Introduction

Reptiles living in the wild are susceptible to various infectious diseases caused by bacteria, fungi, viruses, and parasites [1]. These diseases are known to cause morbidity and mortality in both captive and wild populations of reptiles, including Testudines. When captive reptiles come in contact with specimens from the wild, infection can be transmitted through the shedding of bacteria when the animals defecate in the surrounding environment. Inadequate health management of captive collections makes them a significant source of infectious agent transmission to the captive population, domestic animals, and humans [2]. Moreover, inadequate response often reflects a lack of knowledge of efficient treatment.

Although many different types of bacteria have been isolated from different reptile lesions, most of the time, these isolates, whether from superficial tissue, internal tissues, orifices, or excretory products, had disease-causing organisms among them. While reptiles can get infected with Gram-positive bacteria, most of the causative agents identified from sick reptiles are Gram-negative bacteria [3] such as *Aeromonas*, *Pseudomonas*, *Salmonella*, *Proteus*, *Klebsiella*, *Citrobacter*, *Enterobacter*, *Escherichia*, *Morganella*, and *Providencia* species [4].

It was previously determined that Testudines could potentially harbour *E. coli* [5], but the frequency greatly depends on their diet and their contact with other animals [6]. Chelonitoxism is a specific type of seafood poisoning that can occur from consuming the flesh of certain marine turtles [7]. In addition, Turtle fisheries remain a vital source of cash, protein, and cultural identity in certain parts of the world [7]. A recent study examined the occurrence of E. coli O157:H7, a zoonotic enterohaemorrhagic serotype, in Loggerhead sea turtle (*Caretta caretta*) and green turtle (*Chelonia mydas*) located in the Gulf of Taranto [8]. Even though *E. coli* was not detected, this case study does recognize that turtles act as indicators of public health in the marine environment. Pathogenic *E. coli* strains are accountable for approximately 56 million cases of diarrhoea, resulting in 200,000 human deaths annually globally, with the majority of cases occurring in children aged between 2 and 5 years [5].

Based on the virulence characteristics of this Gram-negative bacterium and the specific location of the infection within the human host, pathogenic strains of *E. coli* are categorized into Enteropathogenic *E. coli* (EPEC), Enterohaemorrhagic *E. coli* (EHEC), Enterotoxigenic *E. coli* (ETEC), Enteroaggregative *E. coli* (EAEC), Enteroinvasive *E. coli* (EIEC), Diffusely Adherent *E. coli* (DAEC) and a new pathotype, *Adherent-Invasive E. coli* (AIEC) [9]. The pathogenic strains of *E. coli* that affect host digestive systems are primarily clonal groupings defined by shared O and H antigens, which define serotypes or serogroups [10]. Pathogenic *E. coli* strains can cause several illnesses in both people and animals due to their virulence characteristics. Investigating the genes encoding virulence factors is necessary for determining the harmful nature of the strains and the pathophysiology of infections [9]. Shiga toxin (*stx*) is often produced by *E. coli* O157:H7 and is responsible for haemorrhagic colitis, haemolytic uremic syndrome, and diarrhoea [11]. The *stx* has two subgroups: *stx1* and *stx2*, the *stx2* has more severe consequences than *stx1* [12].

Numerous studies have been conducted to investigate the prevalence of antibiotic-resistant strains of *E. coli* in the intestinal tract of warm-blooded animals [6], including humans, birds [5], and livestock [13]. However, research on the presence and resistance of *E. coli* bacteria in Testudines, whether wild or captive, remains limited [14]. Generally, antibiotic resistance is due to the overuse and abuse of antibiotics, leading to high levels of antibiotic resistance in *E. coli* in human, animal, and human health is a significant public health concern. Consequently, antibiotic-resistant *E. coli* infections can be difficult or impossible to treat, increasing morbidity and mortality [15]. This study sought to present new data on the prevalence of virulence and antibiotic-resistant profile of *E. coli* in a wild Testudine population in South Africa.

## Materials and methods

### Study area and sample collection

Samples were collected near Walkers River Camp, located on the banks of the Klaserie River at the westernmost perimeter of the Timbavati Private Nature Reserve (TPNR). Four species of Testudines are known to occur in TPNR namely: leopard tortoise (*Stigmochelys pardalis*), Speke’s hinged-back tortoise (*Kinixys spekii*), marsh terrapin (*Pelomedusa subrufa)* and serrated hinged terrapin (*Pelusios sinuatus*) [16].

**Fig. 1:**
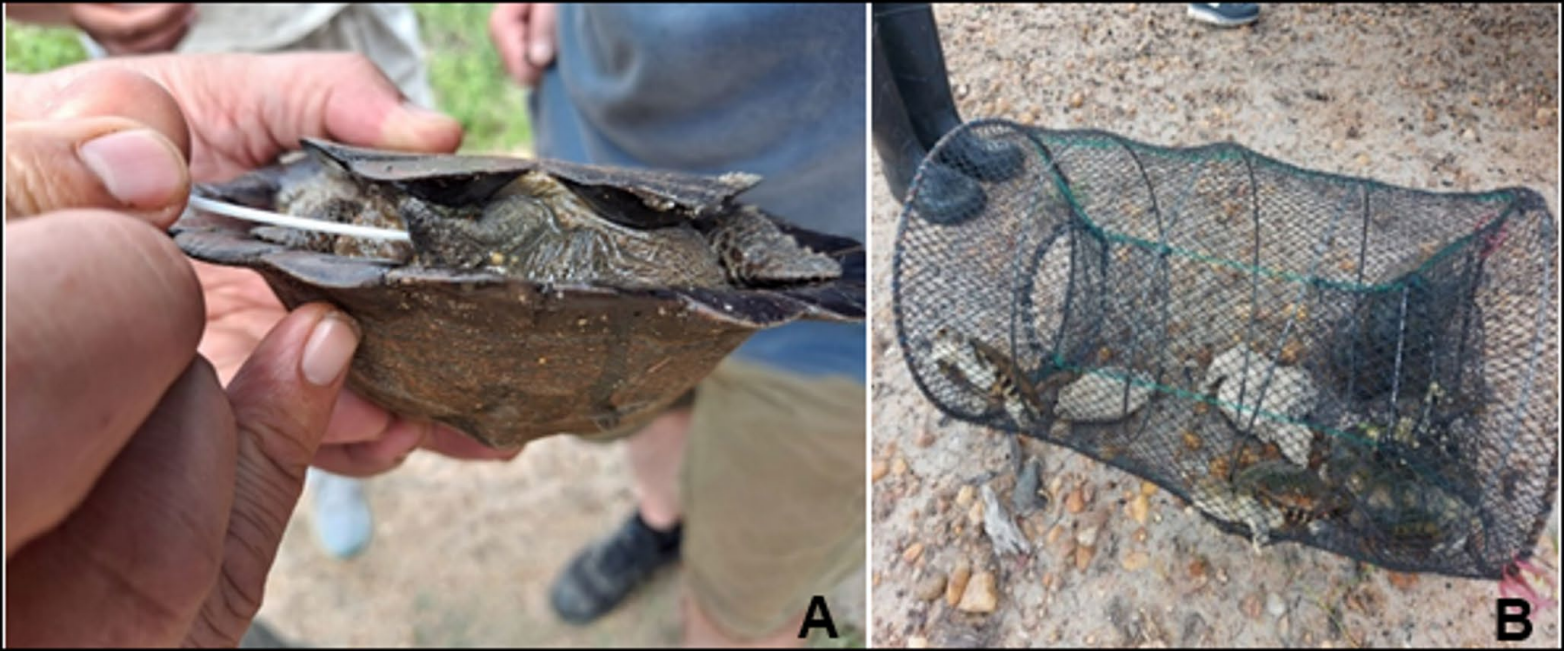
Terrapins being processed for samples **A**: a cloacal swab taken from a *Pelusios sinuatus*. **B**: Terrapins in a funnel trap

Funnel traps were set in areas of high terrapin activity (confirmed basking or breaking of water surface). Traps were baited with chicken livers and placed in a shaded or semi-concealed area, in shallow water, stabilized with metal rods, and attached to a tree branch to prevent accidental displacement [17] (Fig. 1). Traps were inspected after 12 and 24 h, individual specimens were placed in pondwater buckets, sampled, and released at the point of capture. Tortoises were collected through active sampling by road cruising during active morning hours. Spotted tortoises were captured by hand, placed inside a dry bucket for sampling, and promptly released at the point of capture.

Sterile cotton swabs (Rotilabo, Separations) were used to sample the cloaca of terrapins and tortoises. Samples were stored at 4 °C. Aseptic techniques were followed when handling all animals and the working surface was sterilized with 70% ethanol before and after sampling each animal. Sediment samples were also collected from water bodies where terrapins, tortoises, or both were collected. Water bodies were classified as permanent or temporary (retaining water for a few days or weeks after sufficient rain). The sediment was scooped with 2 mL tubes and temporarily stored at 4 °C. Multiple samples were collected from larger permanent water bodies.

### Microbiological techniques and analysis

Buffered peptone water (BPW) was used as the initial enrichment medium for the collected samples. 10 ml of BPW was used to pre-enrich the cloacal swabs and the tubes were incubated at 37 °C with agitation for 24 h. Following inoculation, the samples were cultured on MacConkey agar (MAC) and Eosin Methylene Blue Agar (EMB). Individual colonies were obtained through streak plating. The plates were incubated for 24 h at 37 °C and examined for distinct colonies. *E. coli* colonies on MAC appeared red or pink with non-mucoid texture and were opaque, while on EMB they appeared blue-black with a green metallic sheen, and were circular, smooth and opaque. Plates exhibiting presumed *E. coli* growth were subcultured on EMB and MAC, from which 2–3 colonies were selected for further analysis. All pure *E. coli* isolate colonies were stored in nutrient broth with 15% sterile glycerol at – 80˚C.

### Bacterial genomic DNA extraction

Genomic DNA was extracted from all presumed *E. coli* pure isolates using the traditional boiling method [18]. A volume of 100 µL of distilled water was pipetted into 2 mL microcentrifuge tubes, 2–3 pure colonies were transferred to the tubes, and boiled at 100 °C in a dry warm bath for 15 min. They were then centrifuged at 10 000 rpm for 10 min, and the supernatant was transferred to a new labelled microcentrifuge tube.

### Genetic identification of *E. coli*

All positive controls utilized in this study for identification, virulence genes, and antibiotic resistance genes were sourced from our previous research [19]. PCR was performed to amplify the targeted *uidA* gene (147 bp) (uidA-F: AAA ACG GCA AGA AAA AGC; uidA-R: ACG CGT GGT TAC AGT CTT GCG) for *E. coli* identification [19]. Each PCR reaction consisted of a total reaction mixture of 25 µL consisting of 12.5 µL of 2X DreamTaq Green Master mix (0.4 mM dCTP, 0.4 mM dATP, 0.4 mM dTTP, 0.4 mM dGTP, 4 mM MgCl_2_ and a loading buffer) (ThermoFisher Scientific, South Africa), 8.5 µL of nuclease-free water, 2 µL of the template DNA, and 1 µL of each oligonucleotide primer. The following conditions were used: 94 °C for 2 min, 25 cycles of: 94 °C for 1 min; 60 °C for 1 min; 72 °C for 1 min, and a final extension step at 72 °C for 2 min. All samples which were amplified by PCR were subjected to 2% agarose gel electrophoresis with 100 bp DNA molecular weight marker used to determine the sizes of the DNA fragments. The gel was stained with ethidium bromide (0.5 µg/mL) and viewed under UV light.

Furthermore, *16S rRNA* PCR assay was performed to amplify the targeted *16S rRNA* universal genes 1492R (5’-GGT TAC CTT GTT ACG ACT T-3’) and 27 F (5’-GAG TTT GAT CCT GGC TCA G-3’) [20]. Each PCR reaction consisted of a total reaction mixture of 25 µL consisting of 12.5 µL of 2X DreamTaq Green Master mix (0.4 mM dCTP, 0.4 mM dATP, 0.4 mM dTTP, 0.4 mM dGTP, 4 mM MgCl_2_ and a loading buffer) (ThermoFisher Scientific, South Africa), 2 µL of the template DNA, 8.5 µL of nuclease-free water and 1 µL of each oligonucleotide primer [each at 10 µM concentration]. The following conditions were met: 95 °C for 5 min for, 30 cycles, 95 °C for 5 min, 55 °C for 30 s, 72 °C for 1 min and a final extension step 72 °C for 5 min. All samples which were amplified by PCR were subjected to agarose gel electrophoresis as described above. The PCR products were purified using the QIAquick Gel Extraction Kit (Qiagen, Hilden, Germany), following the manufacturer’s instructions. The purified products were then submitted for sequencing at Inqaba Biotechnological Industries (Pty) Ltd. (Pretoria, South Africa). Sequence visualization and editing were performed using the Molecular Evolutionary Genetics Analysis software (version 7.0, MEGA7). Using BLASTn (http://www.ncbi.nlm.nih.gov/BLAST/), the sequenced *16 S rRNA* gene of the representative isolates was compared to nucleotide sequences found in GenBank and identified by comparing it with those found in the National Center for Biotechnology Information database (NCBI).

### PCR amplification of O-serogroups for *E. coli*

Three different *E. coli* -O-serogroups, including 0103, 0145 and 0157 were screened for using PCR as described by previous studies (Table 1). Each PCR reaction consisted of a total reaction mixture of 25 µL consisting of 12.5 µL of 2X DreamTaq Green Master mix (0.4 mM dCTP, 0.4 mM dATP, 0.4 mM dTTP, 0.4 mM dGTP, 4 mM MgCl_2_ and a loading buffer) (ThermoFisher Scientific, South Africa), 2 µL of the template DNA, 8.5 µL of nuclease-free water and 1 µL of each oligonucleotide primer. All samples which were amplified by PCR were subjected to agarose gel electrophoresis as described above.

**Table 1:** Oligonucleotide primers used for amplification of different targeted genes of different strains of *E. coli* and PCR conditions used in this study

Primer	Sequence	Target gene	Gene Size	PCR conditions	References
O157 F O157 R	CGGACATCCATGTGATATGG TTGCCTATGTACAGCTAATCC	*rfbE*	259	95 °C, 3 min(1x); 95 °C, 20 s; 58 °C, 40 s; 72 °C,30 s (30x); 72 °C for 8 min (1x)	[21]
O145 F O145 R	CCATCAACAGATTTAGGAGTG TTTCTACCGCGAATCTATC	*wzx*	609	95 °C, 3 min(1x); 95 °C, 20 s; 58 °C, 40 s; 72 °C,30 s (30x); 72 °C for 8 min (1x)	[21]
O103 F O103 R	TTGGAGCGTTAACTGGACCT GCTCCCGAGCACGTATAAG	*wzyA*	321	95 °C, 3 min(1x); 95 °C, 20 s; 58 °C, 40 s; 72 °C,30 s (30x); 72 °C for 8 min (1x)	[21]

### Determination of virulence genes of *E. coli*

Various Shiga toxin-producing *E. coli* STEC virulence genes including Shiga toxin type1 (*stx1*) and type 2 (*stx2*), *Vir* and *eaeA* were screened using PCR. Each PCR reaction consisted of a total reaction mixture of 25 µL consisting of 12.5 µL of 2X DreamTaq Green Master mix (0.4 mM dCTP, 0.4 mM dATP, 0.4 mM dTTP, 0.4 mM dGTP, 4 mM MgCl_2_ and a loading buffer) (ThermoFisher Scientific, South Africa), 8.5 µL of nuclease-free water, 2 µL of the template DNA, and 1 µL of each oligonucleotide primer. The PCR conditions are listed in (Table 2). ChipPlot was used to create heat maps for virulence genes (https://www.chiplot.online/#).

**Table 2:** Primers utilized for the amplification of different targeted virulence genes

Pathotypes	Primer	Sequence	Target gene	Gene size	PCR conditions	Reference
**STEC**	Stx1-F Stx1-R	AAATCGCCATTCGTTGACTACTTCT TGCCATTCTGGCAACTCGCGATGCA	*stx1*	366	95 °C, 3 min(1x); 95 °C, 1 min; 55 °C, 1 min; 72 °C, 1 min (35x); 72 °C for 10 min (1x)	[21]
**STEC**	Stx2-F Stx2-R	CGATCGTCACTCACTGGTTTCATCA GGATATTCTCCCCACTCTGACACC	*stx2*	282	95 °C, 3 min(1x); 95 °C, 1 min; 55 °C, 1 min; 72 °C, 1 min (35x); 72 °C for 10 min (1x)	[21]
**EIEC**	EaeA-F EaeA-R	TGCGGCACAACAGGCGGCGA CGGTCGCCGCACCAGGATTC	*eaeA*	629	94 °C, 5 min(1x); 94 °C, 1 min; 55 °C, 30 s; 72 °C, 1 min (35x); 72 °C for 5 min (1x)	[21]
**EIEC**	Vir-F Vir-R	AGCTCAGGCAATGAAACTTTGAC TGGGCTTGATATTCCGATAAGTC	*Vir*	607	94 °C, 5 min(1x); 94 °C, 1 min; 54 °C, 30 s; 72 °C, 1 min (35x); 72 °C for 5 min (1x)	[21]

STEC; Shiga toxin-producing *Escherichia coli*, and EIEC; enteroinvasive *E coli*

### Antimicrobial susceptibility test

Antimicrobial susceptibility was performed using the disc diffusion method on Mueller-Hinton agar (Merck, Germany). The antibiotics tested included Nalidixic acid (NA) (30 µg), Streptomycin (S) (10 µg), Gentamicin (CN) (10 µg), Ciprofloxacin (CIP) (5 µg), Tetracycline (TE) (30 µg), Erythromycin (E) (15 µg), Cephalothin (KF) (30 µg), Spectinomycin (SH) (10 µg), (ThermoFisher Scientific™, South Africa). These antibiotic susceptibility discs were positioned several centimetres apart on the plates and were then incubated at 37 °C for 24 h in a non-inverted position. The results were observed and recorded according to the Clinical and Laboratory Standards Institute (CLSI) guidelines [22]. Colistin susceptibility was determined by the broth dilution method as recommended by the CLSI [23]. CLSI guidelines recommend a clinical resistance breakpoint for colistin as greater than or equal to ≥ 2 µg/ml [24]. For quality control purposes, *Escherichia coli* ATCC 25,922 was utilized in the antimicrobial susceptibility test. Isolates were classified as multidrug-resistant (MDR) if it was resistant to three or more classes of antibiotics.

### Determination of antibiotic-resistant genes of *E. coli* isolates

Antibiotic resistance genes from four different classes, Quinolones (*qnrA*,* qnrD* and *qnrS*), colistin (*mcr-1*, *mcr-2*, and *mcr-4*) Tetracycline (*tetW*, *tetA* and *tetO*), beta-lactamase genes (*bla*_TEM_, *bla*_SHV_ and *bla*_OXA_) and Integrase class 1 (*Intl 1*) were screened on positive *E. coli* samples using PCR (Table 3). Each PCR reaction consisted of a total reaction mixture of 25 µL consisting of 12.5 µL of 2X DreamTaq Green Master mix (0.4 mM dCTP, 0.4 mM dATP, 0.4 mM dTTP, 0.4 mM dGTP, 4 mM MgCl_2_ and a loading buffer) (ThermoFisher Scientific, South Africa), 2 µL of the template DNA, 8.5 µL of nuclease-free water and 1 µL of each oligonucleotide primer.

**Table 3:** Oligonucleotide primers used for amplification of different targeted antibiotic-resistant genes

Primer	Sequence	Target gene	Gene Size	PCR conditions	References
tetA-F tetA-R	GCGCTNTATGCGTTGATGCA ACAGCCCGTCAGGAAATT	*tet(A)*	387	94 °C, 6 min(1x); 94 °C, 30 s; 62 °C, 30 s; 72 °C, 1 min (30x); 72 °C for 6 min (1x)	[25]
tetO-F tetO-R	ACGGARAGTTTATTGTATACC TGGCGTATCTATAATGTTGAC	*tet(O)*	171	94 °C, 6 min(1x); 94 °C, 30 s; 60 °C, 30 s; 72 °C, 1 min (30x); 72 °C for 6 min (1x)	[25]
tetW-F tetW-R	GAGAGCCTGCTATATGCCAGC GGGCGTATCCACAATGTTAAC	*tet(W)*	168	94 °C, 6 min(1x); 94 °C, 30 s; 50 °C, 30 s; 72 °C, 1 min (30x); 72 °C for 6 min (1x)	[25]
SHV-F SHV-R	CACTCAAGGATGTATTGT G TTAGCGTTGCCAGTGCTCG	*bla*_SHV_	885	94 °C for 5 min, 94 °C for 45 s, 55 °C for 30 s, 72 °C for 60 s, 72 °C for 10 min.	[25]
OXA-F OXA -R	ACACAATACATATCAACTTCGC AGTGTGTTTAGAATGGTGATC	*bla*_OXA_	813	94 °C for 5 min, 94 °C for 45 s, 55 °C for 30 s, 72 °C for 60 s, 72 °C for 10 min.	[25]
TEM-F TEM-R	TTC TTG AAG ACG AAA GGG C ACGCTCAGTGGAACGAAAAC	*bla*_TEM_	1150	94 °C for 5 min, 94 °C for 45 s, 55 °C for 30 s, 72 °C for 60 s, 72 °C for 10 min.	[25]
qnrA-F qnrA-R	ATTTCTCACGCCAGGATTTG GAGATTGGCATTGCTCCAGT	*qnrA*	413	95 °C, 5 min(1x); 94 °C, 1 min; 56 °C, 1 min; 72 °C, 1 min (35x); 72 °C for 10 min (1x)	[26]
qnrD-F qnrD-R	GCTGGAGCTTGTCAGGGATT TGCTGCGAGATATCATGCGT	*qnrD*	585	95 °C, 5 min(1x); 94 °C, 1 min; 59 °C, 1 min; 72 °C, 1 min (35x); 72 °C for 10 min (1x)	[26]
qnrS-F qnrS-R	CCCCATGCCCGAAGTTATCA ACTGCTTGGAGTGTGTTGGT	*qnrS*	457	95 °C, 5 min(1x); 94 °C, 1 min; 59 °C, 1 min; 72 °C, 1 min (35x); 72 °C for 10 min (1x)	[26]
Intl1-F Intl1-R	GCCTTGCTGTTCTTCTACGG GATGCCTGCTTGTTCTACGG	*Int1*	558	94 °C, 5 min(1x); 94 °C, 30 s; 55 °C, 30 s; 72 °C, 2 min (35x); 72 °C for 5 min (1x)	[25]
mcr-1-F mcr-1-R	TATCGCTATGTGCTAAAGCCTG CGTCTGCAGCCACTGGG	*mcr-1*	1139	94 °C, 5 min(1x); 94 °C, 30 s; 56 °C, 1 min; 72 °C, 1 min (25x); 72 °C for 5 min (1x)	[27]
mcr-2-F mcr-2-R	TATCGCTATGTGCTAAAGCCTG AAAATACTGCGTGGCAGGTAGC	*mcr-2*	816	94 °C, 5 min(1x); 94 °C, 30 s; 56 °C, 1 min; 72 °C, 1 min (25x); 72 °C for 5 min (1x)	[27]
mcr-4-F mcr-4-R	ATCCTGCTGAAGCATTGATG GCGCGCAGTTTCACC	*mcr-4*	405	94 °C, 5 min(1x); 94 °C, 30 s; 56 °C, 1 min; 72 °C, 1 min (25x); 72 °C for 5 min (1x)	[27]

## Results

### Identification of *E. coli* through the amplification of the *UidA* gene

A total of 20 sediment samples (5 permanent water bodies and 15 temporary water bodies) and 36 wild Testudines were sampled, including *Stigmochelys pardalis* (12; 33.3%), *Pelusios sinuatus* (16; 44.4%), *Kinixys spekii* (1; 2.7%) and *Pelomedusa subrufa* (7; 19.4%). A total of 87 presumptive *E. coli* isolates were screened for the presence of the *uidA* gene and 54 (62%) isolates (*n* = 23 Testudines and *n* = 31 sediments) were confirmed to be *E. coli* because of the amplification of the *uidA* gene.

### Nucleotide sequence identity using *16 S rRNA*

The *uidA* gene-positive isolates were subjected to *16 S rRNA* gene analysis for further identification. Representative *E. coli* isolates from sediment and wild Testudines, which underwent sequencing, exhibited a high degree of nucleotide similarity (97.8–99.4%) to *E. coli* reference sequences deposited in GenBank. The representative isolates were deposited in GenBank under the following accession numbers: PP952123, PP952124, PP952125 and PP952126.

### PCR detection of serogroups and virulence genes from *E. coli* isolates

**Fig. 2:**
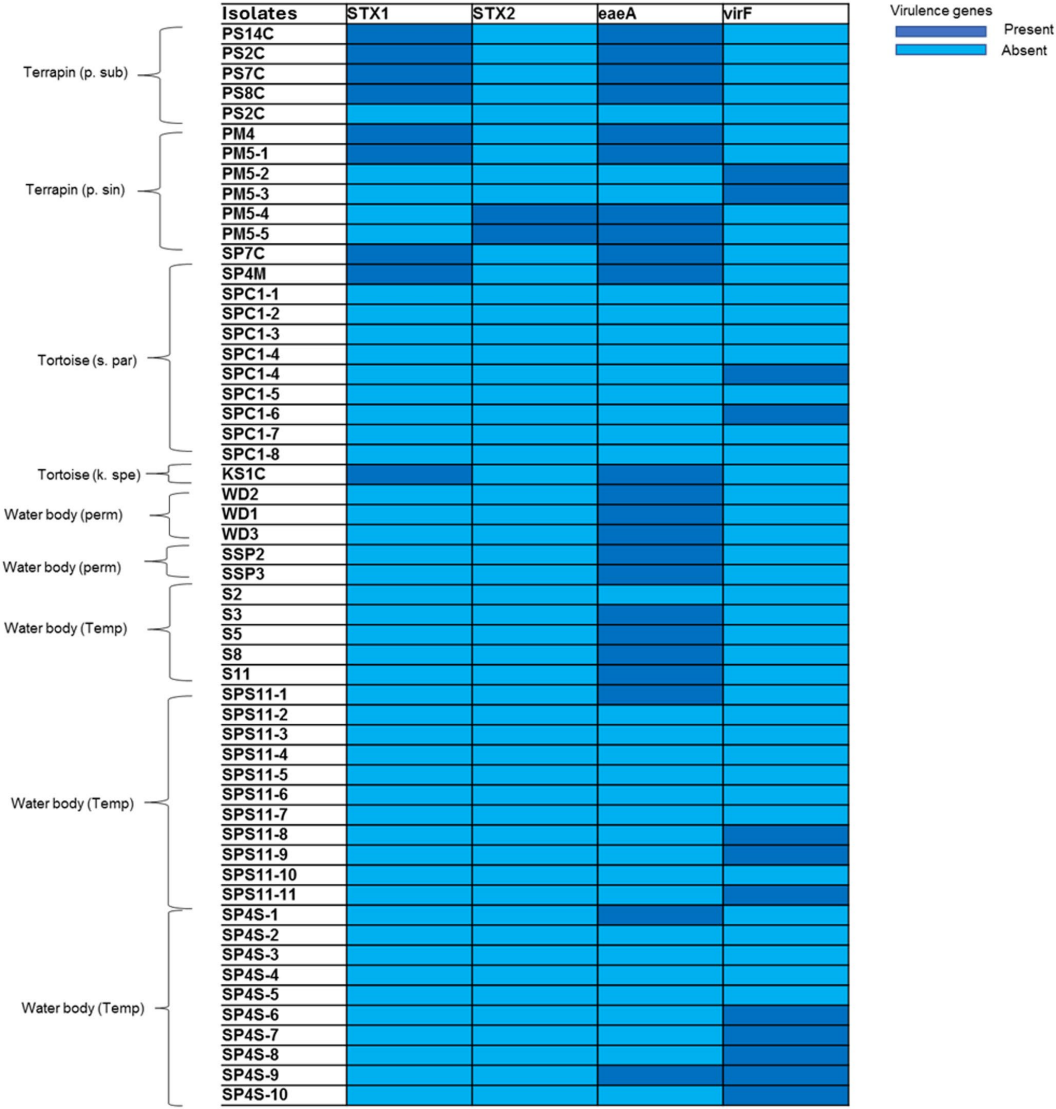
Heatmap of the presence and absence of four virulence genes from the *E. coli* isolated from wild Testudines (https://www.chiplot.online/)

All four of the virulence genes that were screened in this study (*eaeA*, *virF*, *stx1*, and stx2) were successfully detected in the *E. coli* isolates, although in various frequencies. The *eaeA* gene was significantly more prevalent (23/54, 42%) (*n* = 11 samples from Testudines and *n* = 12 from sediments), followed by *virF* (12/54, 22%) (4 samples from Testudines and 8 samples from sediments), *stx2* (2/54, 3%) (2 samples from Testudines) and *stx1* (9/54, 16%) (9 samples from Testudines) (Fig. 2). High rate of isolates (16.7%) (Testudines) carried a combination of *stx2* + *stx1*, whilst 3.7% (Testudines) had a combination of *stx2* + *eaeA*, and 1.9% (Testudines) harboured a mix of *stx1* + *eaeA* genes. The isolates were further screened for the presence of O-serogroups *E. coli* 103, *E. coli* 0145, and *E. coli* 0157, but none of the isolates (Testudines and sediments) tested positive for any of these three serotypes. A total of 26 isolates were classified as Shiga toxin-producing *E. coli* (STEC) as they carried the *stx1* and/or *stx2* and *eaeA* genes.

### Antibiotic resistance profile of *E. coli* isolates

**Fig. 3:**
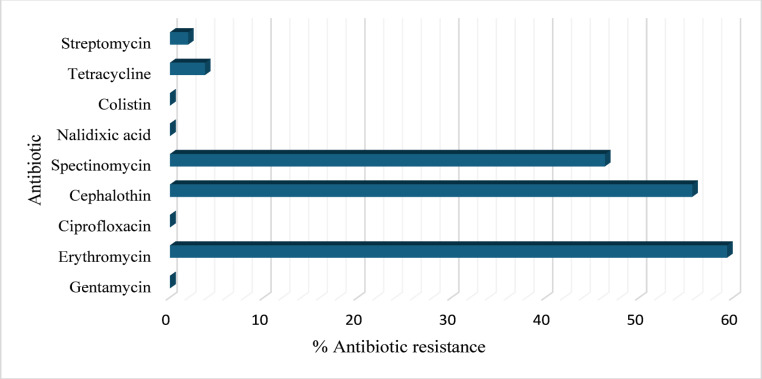
Percentage of resistant *E. coli* isolates identified in this study

All the *E. coli* isolates (Testudines and sediments) were susceptible to gentamycin, ciprofloxacin, nalidixic acid, and colistin (Fig. 3). The isolates exhibited the highest level of resistance to erythromycin (32/54; 59.3%) of half was from Testudines and another half from sediment samples, followed by cephalothin (30/54; 55.6%) of which 14 were from Testudines and 16 from sediment samples, spectinomycin (25/54; 46.3%) of which 15 was from Testudines and 10 from sediment samples, tetracycline (2/54; 3.7%) was from Testudines samples, and lastly streptomycin (1/54; 1.9%) was a Testudines sample. Although 53.7% of the tested strains were resistant to more than one antibiotic, none of the *E. coli* isolates demonstrated multidrug resistance. None of the isolates showed resistance to colistin, gentamicin, nalidixic acid, or ciprofloxacin.

### Characterization of antibiotic resistance genes in *E. coli* isolates

**Fig. 4:**
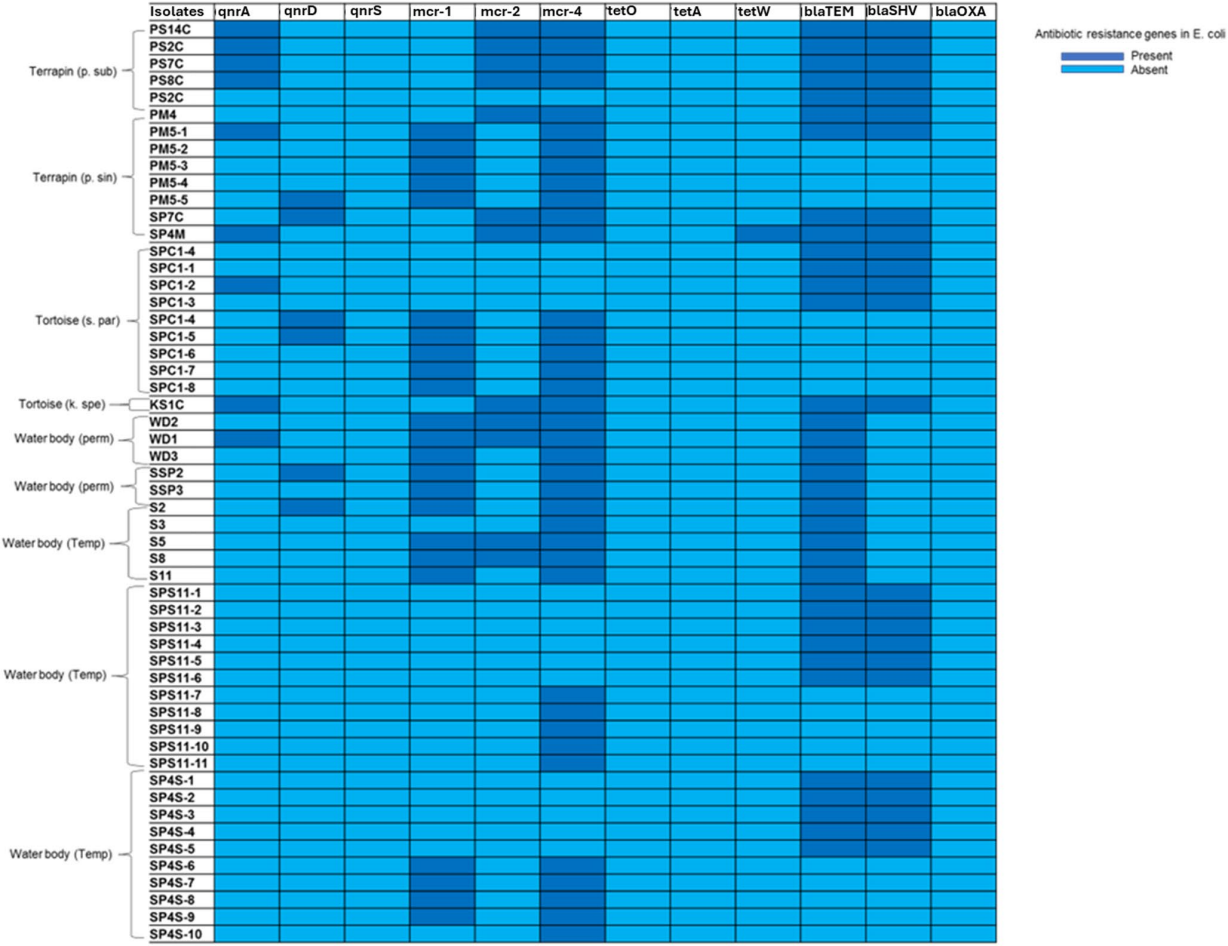
Heatmap of the frequency of 12 different antimicrobial resistance genes from the *E. coli* isolated from wild Testudines

All the 54 *E. coli* isolates were screened for 12 different antimicrobial resistance genes from four different classes of antibiotics. For the quinolones class, it was observed that some isolates possessed the *qnrA* gene (9/54; 16%) of which 8 were from Testudines and 1 from a sediment sample, followed by *qnrD* (6/54, 11%) of which 4 were from Testudines and 2 from sediment samples, while none of the isolates possessed the *qnrS* gene. For colistin, the *mcr-1* gene was present in 23/54 (42%) of the isolates (10 from Testudines and 13 sediment) and 12/54 (22%) of isolates (8 Testudines and 4 sediment) were positive for *mcr-2* gene, while, the *mcr-4* gene was prevalent, detected in 38 out of 54 samples (70%). In the tetracycline class, the *tetO* and *tetA* genes were not detected in any of the isolates, while *tetW* gene was detected from single isolate from Testudines. In the beta-lactamase class, 35/54 (64%) isolates (14 from Testudines and 21 from sediment samples) possessed the *bla*_TEM_ gene, whilst 25/54 (46%) isolates (14 from Testudines and 11 from sediment samples) possessed the *bla*_SHV_ gene and none of the isolates possessed the *bla*_Oxa_ gene (Fig. 4). Of the 54 isolates, 25 (46%) harbored a combination of the *bla*_TEM_ and *bla*_SHV_ genes.

## Discussion

The study identified *E. coli* in 62% of the presumptive isolates from Testudine cloacal (26%) and sediment samples (35.6%) collected from the Timbavati Private Nature Reserve (TPNR). The high recovery rate of *E. coli* may be attributed to the persistence of the bacterium in the host and the environment. As noted by Gordon & Cowling [28], factors such as exposure frequency, colonization duration, and bacterium persistence are key in establishing *E. coli* populations in hosts, which are crucial when assessing the impacts of human-wildlife interactions. Previous studies on reptiles, particularly captive species, have indicated a prevalence of *E. coli* ranging from 44 to 100% [5, 29].

Most research on *E. coli* in wild reptiles has focused on the O157 serogroup [30], which is known for its zoonotic potential and frequent presence in mammals. Conversely, in this study, none of the isolates tested positive for O-serogroups screened, including O157 serotypes. Recent studies on reptiles mainly focus on *E. coli* isolates without detailed molecular characterization, including screening for virulence genes like *eaeA*, which are more commonly studied in mammals and birds [31]. This represents a significant gap in modern-day research, which this study aimed to address, especially considering the potential zoonotic risks posed by reptiles [32].

In our findings, we observed a 42% prevalence of *eaeA* in the samples, with 20% originating from Testudines and 22% from sediment samples. According to research, the prevalence of the *eaeA* gene is notably higher in captive animals compared to wild animals, ranging from 46 to 100% [33], while prevalence in wild animals ranges from 10 to 45.2% [34, 35]. Previous environmental studies have reported a low prevalence of *E. coli* containing the *eaeA* gene, between 27.6% and 29.44% [34, 36]. These virulent strains typically belong to the B1 phylogenetic group, which is commonly associated with animals and environmental sources.

In this investigation, Shiga toxin-producing *Escherichia coli* (STEC) strains carrying the *stx1* gene were identified in 16% of the analyzed isolates, while those harboring the *stx2* gene were detected in only 3% of the isolates. The co-expression of *stx1*, *stx2*, and *eaeA* genes has been suggested to enhance the pathogenic potential of *E. coli* strains [37]. Notably, Jajarmi et al. [37] identified the *stx1* + *stx2* genotype in one-fifth of caprine isolates, while Ramatla et al. [26] observed a higher prevalence of 43.4% from small stock (sheep and goats). Additionally, *stx2* + *eaeA* was detected in 3.7% of isolates, and *stx1* + *eaeA* in 1.9% in the current study. The low occurrence of these gene combinations aligns with findings from Karama et al. [38], who reported on only a single isolate having both *stx2* and *eaeA* genes. Other studies have shown a higher prevalence of *stx1* compared to *stx2* in captive reptiles and mammals [29]. Conversely, studies in wild animals have also demonstrated a higher prevalence of *stx2* compared to *stx1* [39]. To our knowledge, this is the first study investigating the presence and prevalence of *stx* in wild reptiles. The presence of enteroinvasive *E. coli* (*virF* gene) was detected in 22% of the samples in the current study. This conforms to a study conducted in South Africa by Ramatla et al. [26] on small stock (sheep and goats) that detected 18.9% of enteroinvasive *E. coli* (*vir*).

Environmental antibiotic resistance is a global concern, with the environment acting as a reservoir for AMR and contributing to the spread of resistant strains [40]. In this study, *E. coli* isolates from Testudine samples exhibited high phenotypic resistance to three of the antimicrobial agents tested, namely, erythromycin, cephalothin and spectinomycin. The resistance to erythromycin, cephalothin, spectinomycin tetracycline, and streptomycin aligns with previous studies, which exhibited resistance to these antibiotics in *E. coli* isolated from reptiles [41]. Other studies have shown antibiotic resistance in the same antibiotics used in this study from *E. coli* isolated from mammals [42, 43].

Antimicrobial resistance in wildlife does not necessarily indicate direct transmission from humans [44], as HGT is common in high microbial density areas [44], allowing microorganisms to transfer resistance determinants. This study revealed an absence of *tetO* and *tetA* genes, with *tetW* present in only one sample. Previous research on *E. coli* from farmed poultry has reported *tetA* as the most common tetracycline resistance gene among isolates [45]. Conversely, research in other settings has documented the presence of *tetA*, *tetO*, and *tetW* genes in livestock [27]. Quinolones are a class of synthetic, broad-spectrum antimicrobials that inhibit DNA supercoiling and ultimately cause DNA strand breaking by targeting bacterial DNA gyrase (topoisomerase II) and topoisomerase IV []. In this study, the detection of *qnrA* and *qnrD* genes, coupled with the absence of *qnrS*, emphasizes regional and host variations (terrapins and tortoises) in the prevalence of these genes. Identifying *qnr* in *E. coli* isolated from reptiles suggests that these animals may contribute to the environmental dissemination of quinolone-resistant bacteria. Such findings emphasize the importance of enhanced surveillance for antibiotic resistance within non-traditional hosts to reduce zoonotic transmission risks to humans. Isolates carrying qnr determinants, as well as other plasmid-mediated quinolone resistance genes, exhibit phenotypic susceptibility to quinolones/fluoroquinolones in vitro. However, the presence of these genes can confer resistance in vivo [46].

In the present study, we observed a significant prevalence of beta-lactamase genes among the *E. coli* isolates, with 64% harboring the *bla*_TEM_ gene and 46% possessing the *bla*_SHV_ gene. Notably, none of the isolates tested positive for the *bla*_OXA_ gene. Comparative analysis with other studies reveals both similarities and differences. For example, Ranjbar and Sami [47] reported a lower prevalence of the *bla*_TEM_ (37%) and *bla*_SHV_ (27%) genes compared to our findings of 64% and 46%, respectively. Interestingly, they detected the *bla*_OXA_ gene in 25% of isolates, contrasting with our results, which indicated the absence of this gene in the isolates obtained from water sources. This study has some limitations. Notably, the isolates were not screened for ESBL or simple penicillinase variants, which could have provided further insights.

In a separate study, Islam et al. [48] reported a notably higher prevalence of the *bla*_TEM_ gene (90.48%) than our findings of 64%, while the prevalence of *bla*_SHV_ (42.86%) was consistent with our observation of 46%. The differences in *bla*_TEM_ prevalence may be attributed to differences in host species or environmental factors, as the focus of the study by Islam et al. [48] was on migratory birds. Additionally, Gundran et al. [49] found the *bla*_TEM_ gene in 57.97% of their *E. coli* isolates from chicken slightly lower than the 64% observed in our study, while *bla*_SHV_ was detected in only 27.54% of their isolates, significantly less than the 46% we identified among isolates from broiler farms. Conversely, Karczmarczyk et al. [50] reported a high prevalence of *bla*_TEM_ (89.2%), considerably exceeding our findings, while their detection of *bla*_SHV_ (6.8%) was much lower than 46% recorded in the current study.

One of the most crucial antimicrobial medicines in veterinary medicine is colistin, an antibiotic from the polymyxin family. In our study, we observed a high prevalence of colistin resistance genes whereby *mrc1* gene was present in 42% of the isolates, 22% were positive for *mrc2* gene and 70% of the isolates harboured *mrc4* gene. Notably, in this study *E. coli* isolates demonstrated susceptibility to colistin despite possessing *mcr-1*, *mcr-2*, and *mcr-4* genes. This discrepancy may result from the silent expression of resistance genes, transient phenotypic alterations, or the existence of resistance mechanisms that are not fully manifested under the tested conditions [51].

Identifying the plasmid-mediated *mcr-1* gene, which confers colistin resistance, presents a considerable threat to the effectiveness of polymyxins and has emerged as a growing global concern. The *mcr-1* gene has been detected in a diverse array of *Enterobacteriaceae* species across more than 50 countries, originating from various environments, including food, humans, livestock, wildlife, rivers, and vegetables [52].

Humans and wild animal species will live in close or overlapping proximities in future because of habitat fragmentation and an expanding human population [53]. In order to completely comprehend wildlife’s role in the ecology of antibiotic resistance and, ideally, create collaborative solutions to solve this pressing global issue, animals such as wild Testudines must be included in future research and monitoring projects. Future studies should also assess impact of bacterial infections on these wild Testudines,

## Conclusions

This study provides the first documented evidence of Shiga toxin-producing *E. coli* (STEC) strains in wild reptiles and their habitats in South Africa. This study supports the hypothesis that wild animals may serve as significant carriers of microbial resistance and virulence determinants that threaten public health. Our findings underscore that AMR remains a continuously evolving challenge, largely driven by the selective pressure exerted by antimicrobial usage. A major public health concern is the high percentage of the *mcr* and *stx* genes found in *E. coli*. Implementing a “One Health” strategy and creating practical plans to stop and manage this possible global health emergency requires an understanding of AMR issues from wildlife.

## Data Availability

No datasets were generated or analysed during the current study.

## Abbreviations

AMR: Antimicrobial resistance
BPW: Buffered peptone water
CLSI: Clinical and Laboratory Standards Institute
DAEC: Diffusely adherent E. coli
DNA: Deoxyribonucleic acid
E. coli: Escherichia coli
EAEC: Enteroaggregative E. coli
EHEC: Enterohaemorrhagic E. coli
EIEC: Enteroinvasive E. coli
EMB agar: Eosin methylene blue agar
EPEC: Enteropathogenic E. coli
ETEC: Enterotoxigenic E. coli
HGT: Horizontal gene transfer
MDR: Multi-drug resistance
PCR: Polymerase chain reaction
Stx: Shiga toxin
TPNR: Timbavati Private Nature Reserve
UV light: Ultraviolet light
WHO: World Health Organization
